# Effectiveness of the SpineCor treatment for large scoliotic curves compared to moderate and small curves

**DOI:** 10.1186/1748-7161-7-S1-O25

**Published:** 2012-01-27

**Authors:** C Coillard, A Circo, C Rivard

**Affiliations:** 1 Sainte Justine Hospital, Montreal, Canada

## Background

The purpose of this retrospective cohort study was to evaluate the effectiveness of the Dynamic SpineCor brace for large adolescent idiopathic scoliosis (40°-50°) compared to moderate (30°-40°) and small curves (15°-30°) [1,2].

## Materials and methods

657 consecutive scoliotic patients that accepted the treatment and had a definite outcome were included in this study. We divided the patients in tree groups depending on the initial Cobb angle: 15-29° (n=378), 30-39° (n=207) and 40-50° (n=72).

Assessment of brace effectiveness included; 1) percentage of patients who have 5° or less curve progression and the percentage of patients who have 6° or more progression at skeletal maturity, 2) percentage of patients who have had surgery recommendation before skeletal maturity.

## Results

Success of the treatment (stabilisation or correction) was achieved in 80.8% of patients with small curves compared to 62.9% for moderate and 46% for large curves. Progression of curves was observed in 14% of small curves compared with 28.9% for moderate and 48.5% for large curves. Two years follow-up post treatment 24.2% (for small), 17% (moderate) and respectively 16% (large) of patients that finished the treatment still corrected their Cobb angle without wearing the brace.

## Conclusions

The SpineCor brace is effective for the treatment of large adolescent idiopathic scoliosis comparing with moderate curves. Moreover, the positive outcome appears to be maintained in the long term.
